# Pre-operative external ventricle drainage improves neurological outcomes for patients with traumatic intracerebellar hematomas

**DOI:** 10.3389/fneur.2022.1006227

**Published:** 2022-10-18

**Authors:** Bao Wang, Li Gao, Yu Zhang, Ming-ming Su, Wei Shi, Yue Wang, Shun-nan Ge, Gang Zhu, Hao Guo, Fei Gao, Ying-Wu Shi, Wen-xing Cui, Zhi-hong Li, Yan Qu, Xue-Lian Wang

**Affiliations:** ^1^Department of Neurosurgery, Tangdu Hospital, Fourth Military Medical University, Xi'an, China; ^2^Department of Neurosurgery, PLA 960^th^ Hospital, Jinan, China; ^3^Department of Health Statistics, School of Public Health, The Forth Military Medical University, Xi'an, China

**Keywords:** traumatic intracerebellar hematoma, external ventricular drainage, hydrocephalus, neurological outcomes, Glasgow Outcome Scale

## Abstract

**Objectives:**

Traumatic intracerebellar hematoma (TICH) is a very rare entity with a high morbidity and mortality rate, and there is no consensus on its optimal surgical management. In particular, whether and when to place external ventricle drainage in TICH patients without acute hydrocephalus pre-operation is still controversial.

**Methods:**

A single-institutional, retrospective analysis of total of 47 TICH patients with craniectomy hematoma evacuation in a tertiary medical center from January 2009 to October 2020 was performed. Primary outcomes were mortality in hospital and neurological function evaluated by GOS at discharge and 6 months after the ictus. Special attention was paid to the significance of external ventricular drainage (EVD) in TICH patients without acute hydrocephalus on admission.

**Results:**

Analysis of the clinical characteristics of the TICH patients revealed that the odds of use of EVD were seen in patients with IVH, fourth ventricle compression, and acute hydrocephalus. Placement of EVD at the bedside can significantly improve the GCS score before craniotomy, as well as the neurological score at discharge and 6 months. Compared with the only hematoma evacuation (HE) group, there is a trend that EVD can reduce hospital mortality and decrease the occurrence of delayed hydrocephalus, although the difference is not statistically significant. In addition, EVD can reduce the average NICU stay time, but has no effect on the total length of stay. Moreover, our data showed that EVD did not increase the risk of associated bleeding and intracranial infection. Interestingly, in terms of neurological function at discharge and 6 month after the ictus, even though without acute hydrocephalus on admission, the TICH patients can still benefit from EVD insertion.

**Conclusion:**

For TICH patients, perioperative EVD is safe and can significantly improve neurological prognosis. Especially for patients whose GCS dropped by more than 2 points before the operation, EVD can significantly improve the patient's GCS score, reduce the risk of herniation, and gain more time for surgical preparation. Even for TICH patients without acute hydrocephalus on admission CT scan, EVD placement still has positive clinical significance.

## Introduction

Compared with traumatic brain injury, traumatic intracerebellar hemorrhage (TICH) has only a few small series of cases reported (1–5). Although TICH is a rare entity, it is associated with high morbidity and mortality. Compared with TICH, spontaneous cerebral hemorrhage has a wider agreement on its management. According to the guidelines for the management of spontaneous intracerebral hemorrhage, those patients with spontaneous cerebellar hemorrhage who are experiencing deterioration in neurological functioning or who are suffering from brainstem compression or hydrocephalus due to ventricular obstruction should undergo immediate surgical removal of the hemorrhage (Class I; Level of Evidence B). It is not recommended to treat these patients initially with ventricular drainage rather than surgical evacuation (Class III; Level of Evidence C) (6). Given the lack of sufficient experience of individual neurosurgeons or a single medical center in the management of TICH, there is an urgent need to explore the optimal treatment strategy for it.

The current surgical methods for TICH mainly include craniotomy, minimally invasive hematoma aspiration, or external ventricular drainage (EVD) only (7). The guidelines on craniocerebral trauma suggest that different surgical plans should be flexibly adopted based on the amount and location of the hematoma, combined with the patient's consciousness and general condition (8). There is no consensus on its optimal surgical management. It remains unclear whether the preoperative placement of EVD is beneficial for neurological outcomes in TICH patients.

It is generally believed that if the patient has acute obstructive hydrocephalus or large ventricular hemorrhage on admission, pre-operation external ventricular drainage (EVD) is required (8). However, for patients whose admission CT showed no acute hydrocephalus, due to the physiological characteristics of the posterior fossa, the probability of hydrocephalus after admission was high and can be progressed quickly (9). Once hydrocephalus has occurred, it is probable that the patient will miss the best opportunity to rescue themselves by placing an EVD. However, if the EVD is placed in advance, it may increase the risk of infection and supratentorial hemorrhage (10–13). Therefore, there is still debate about when to place the EVD on TICH. Therefore, for patients without hydrocephalus on admission CT scan, the significance and indications of EVD placement are worth exploring.

The present study retrospectively evaluated 47 TICH patients surgically managed in a single tertiary medical center to determine whether the preoperative placement of EVD is beneficial for neurological outcomes in TICH patients, with special attention paid to the significance of EVD in TICH patients without acute hydrocephalus on admission CT scan.

## Materials and methods

### Study population and data source

This study obtained approval from the Ethics Committee of our Hospital (TDLL-202206-02) and informed consent of their legal guardians. A total of 47 consecutive TICH patients surgically managed in a tertiary care academic hospital (Tangdu hospital, affiliated with the Air Force Military Medical University) from January 2009 to October 2021 were enrolled in the present study. Patients with supratentorial lesions that require surgery and those with fatal hemorrhages whose therapy did not aim to treat the condition were excluded from the present study. Medical and nursing records, including demographic information, clinical evaluation, imaging, and procedure notes, were retrospectively reviewed. Discharge reports from the rehabilitation facility as well as standardized telephone interviews were used to evaluate the neurological function at 6 month after the ictus. The consent with ethical clearance for the study was obtained from the patients' surrogate.

### Treatment groups and EVD insertion

All TICH patients were managed according to the Chinese Guidelines for Traumatic Surgery (2015 Edition). A decompressive suboccipital craniectomy on the midline, or ipsilateral to the hematoma, with the evacuation of the hematoma as well as the necrotic hemorrhagic tissue, was performed in all 47 patients.

According to whether EVD was placed before the operation, these patients were divided into 2 groups: (1) hematoma evacuation combined with preoperative EVD placement (HE+EVD), and (2) hematoma evacuation only and no preoperative EVD placement (HE). The decision to place a pre-operation EVD at the bedside was based on the presumed benefit to the patients and was up to the discretion of the attending neurosurgeon after discussion of potential benefits, risks, and possible complications with the patients' surrogates. The placement time of preoperative bedside EVD is usually about 0.5–1 h before surgery, and the volume of external drainage is usually controlled at 20–60 ml. All the EVD was placed into the right frontal horn at the Kocher point unless there is a fracture on the right frontal bone, in which case a left EVD was inserted. Gradual EVD weaning was performed after the patient meets the following conditions: improved neurological examination, no need to continue ICP monitoring, and continuous post-operative CT scans showing that intraventricular blood clearance or obstructive hydrocephalus has improved.

### Study variables and outcomes

The presence of TICH, IVH, fourth ventricle involvement and occlusive hydrocephalus was established by a CT scan on admission. The volume of hematoma was calculated using the ABC/2 method as already described by Kothari (14). Glasgow Coma Scale (GCS) was applied to evaluate the level of consciousness pre-EVD, post-EVD, and before craniectomy. The primary outcomes were in-hospital mortality and neurological function evaluated by GOS at discharge and 6 months after the ictus. An GOS ≥4 was considered a favorable functional outcome as described previously (1). The secondary outcomes of interest mainly include the length of stay in the NICU, the total length of hospital stay, EVD puncture tract bleeding, intracranial infection, pneumonia, and delayed hydrocephalus.

### Statistical analysis

Statistical analyses were conducted with the SPSS statistics software package (IBM Corporation, Armonk, New York, USA). Means with SDs or median with ranges were calculated for continuous variables and counts with percentages for categorical variables. Student's *t*-test was used for continuous data and the Chi-square test for categorical data to compare the demographic and clinical characteristics among patients with and without preoperative EVD. A Wilcoxon rank-sum test was used to compare continuous data that were not normally distributed and fisher exact tests for categorical events whose expected cell count was <5. *P* < 0.05 was considered statistically significant.

## Results

A total of 47 TICH patients was included in this study by retrospective screening and reviewing of their medical records, imaging data, and operative notes. According to whether the EVD was placed before the operation, these patients were divided into 2 groups: (1) hematoma evacuation combined with preoperative EVD placement (HE+EVD), and (2) hematoma evacuation only and no preoperative EVD placement (HE).

The demographic and clinical characteristics are summarized in Table 1. The average age of the patients was 44.6 ± 17.8 years old, of which 59.6% were male. There was no statistical significance in age, gender, hematoma volume, and GCS score between HE+EVD and HE groups. Post-traumatic intracerebellar hemorrhages are often associated with skull fractures. In this case series, 36 (76.6%) patients presented with skull fractures, and the difference in distribution between the 2 groups was not statistically significant. Notably, the odds of use of EVD were seen in patients with IVH (*p* = 0.03), fourth ventricle compression (*p* = 0.02), and acute hydrocephalus (*p* < 0.01).

**Table 1:** The demographic and clinical characteristics of patients with traumatic intracerebellar hemorrhage stratified by surgical strategy (*n* = 47).

	**Overall**	**HE + EVD**	**HE**	***p* value**
Number of patients (%)	47 (100)	29 (61.7)	18 (38.3)	
Male (%)	28 (59.6)	17 (58.6)	11 (61.1)	>0.99
Age	44.6 ± 17.8	43.4 ± 18.3	45.7 ± 13.2	0.64
Volume of hemotoma	17.8 ± 8.1	18.2 ± 7.6	17.4 ± 6.9	0.71
Median GCS (range)	7 (3–15)	7 (3–14)	8 (3–15)	0.83
Skull fracture (%)	36 (76.6)	23 (79.3)	13 (72.2)	0.57
Presence of IVH (%)	17 (36.2)	14 (48.3)	3 (16.7)	0.03*
Fourth ventricle involvement (%)	21 (44.7)	17 (58.6)	4 (22.2)	0.02*
Acute hydrocephalus (%)	15 (31.9)	15 (31.9)	0 (0)	<0.01**
Hypertension	23 (48.9)	11 (37.9)	12 (66.7)	0.08
Diabetes	16 (34.0)	8 (27.6)	8 (44.4)	0.34
Heavy drinking	27 (57.4)	15 (51.7)	12 (66.7)	0.37
Warfarin	11 (23.4)	7 (24.1)	4 (22.2)	>0.99
Any antiplatelet drug	18 (38.3)	11 (37.9)	7 (38.9)	>0.99

HE, hemotoma evacuation; EVD, external ventricle drainage; GCS is defined as the lowest Glasgow coma scale before EVD insertion; * and **, statistically significant.

In Table 2, compared with the time of admission, the GCS scores of the two groups both decreased to a certain extent before operation. However, placement of EVD at the bedside could significantly improve the preoperative GCS score (*p* = 0.02). There was a trend that EVD could reduce hospital mortality (27.6 vs. 38.9%), however, the difference was not statistically significant. Importantly, compared with the HE group, EVD significantly improved the neurological outcome at discharge (*p* = 0.045) and 6 months (*p* = 0.04) after ictus.

**Table 2:** Primary outcomes for traumatic intracerebellar hemorrhage managed surgically with or without EVD.

	**GCS**			**In-hospital mortality (*n*, %)**	**Favorable GOS**≥**4 (*****n*****, %)**	
	**Admission**	**Before EVD**	**Before surgery**		**Discharge**	**6 months**
HE+EVD(*n* = 29)	10.0 ± 3.8	5.3 ± 2.3	8.6 ± 3.3	8 (27.6)	15 (51.7)	17 (58.6)
HE (*n* = 18)	10.4 ± 3.2	~	6.4 ± 2.5	7 (38.9)	4 (22.2)	5 (27.8)
*p* value	ns		0.02*	0.41	0.045*	0.04*

HE, hemotoma evacuation; EVD, external ventricle drainage; GOS, Glasgow Outcome Scale; ns: statistically non-significant.

* Statistically significant.

Table 3 revealed that preoperative EVD could reduce the average NICU stay time (*p* = 0.04, 4.9 ± 5.1 vs. 9.4 ± 6.3), but had no effect on the total length of hospital stay. It seemed that EVD tended to decrease the occurrence of delayed hydrocephalus (4.8 vs. 18.2%), although the difference is not statistically significant. In terms of EVD-related hemorrhage, intracranial infection and pneumonia, there was no statistical difference between the two groups.

**Table 3:** Secondary outcomes for traumatic intracerebellar hemorrhage managed surgically with or without EVD.

	**HE + EVD**	**HE**	***p* value**
Mean length of stay (d)			
NICU, survivors	4.9 ± 5.1	9.4 ± 6.3	0.04*
Hospital, survivors	12.4 ± 8.1	13.7 ± 7.2	0.66
EVD related hemorrhage (*n*,%)	0	~	ns
Intracranial infection (*n*,%)	5 (17.2)	3 (16.7)	>0.99
Pneumonia (*n*,%)	16 (55.2)	7 (38.9)	0.37
Delayed hydrocephalus, survivors (*n*, %)	1 (4.8)	2 (18.2)	0.27

HE, hemotoma evacuation; EVD, external ventricle drainage;

* statistically significant.

Furthermore, we extracted the patients without acute hydrocephalus on admission CT scan for comparison and divided them into two groups: (1) hematoma evacuation combined with preoperative EVD placement (EVD) (*n* = 14), and (2) hematoma evacuation only and no preoperative EVD placement (non-EVD) (*n* = 18) (Table 4). Interestingly, in terms of neurological function at discharge (*p* = 0.01) and 6 month after the ictus (*p* = 0.01), even though they did not have acute hydrocephalus on admission CT scan, the TICH patients could still benefit from EVD insertion. 0 patients in the EVD group had delayed hydrocephalus, while 2 patients (18.2%) in the non-EVD group had delayed hydrocephalus. There was no statistical difference in hospital mortality between the two groups (14.3 vs. 38.9%).

**Table 4:** Outcomes for EVD in traumatic intracerebellar hemorrhage without acute hydrocephalus.

	**EVD** **(*n* = 14)**	**non-EVD** **(*n* = 18)**	***p* value**
In-hospital Mortality (*n*, %)	2 (14.3)	7 (38.9)	0.23
Favorable GOS≥4 (*n*, %)			
Discharge	10 (71.4)	4 (22.2)	0.01*
6 months	11 (78.6)	5 (27.8)	0.01*
Delayed hydrocephalus, survivors (*n*, %)	0	2 (18.2)	0.21

HE, hemotoma evacuation; EVD, external ventricle drainage; GOS, Glasgow Outcome Scale.

* Statistically significant.

## Discussion

In this retrospective study, we examined the impact of EVD placement before craniectomy on in-hospital mortality and neurological function for patients with TICH. Our data revealed that preoperative EVD at the bedside can significantly improve the preoperative GCS score, ameliorate the neurological score at discharge and 6 months after the ictus, and reduce the average NICU stay time, without increasing the risk of associated bleeding and intracranial infection. In addition, there is a trend that EVD can reduce in-hospital mortality and decrease the occurrence of delayed hydrocephalus. Interestingly, in terms of neurological function at discharge and 6 month after the ictus, even though without acute hydrocephalus, the TICH patients can still benefit from EVD insertion.

The application of EVD in severe craniocerebral trauma, aneurysmal subarachnoid hemorrhage, and intraventricular hemorrhage has been reported and has achieved good clinical results (15–18). Especially for patients whose intracerebral hematoma broke into the ventricular system, placing EVD can significantly improve the patient's prognosis (18, 19). However, there are few reports on the application of EVD in TICH.

Consistent with previous literature reports, the current study revealed the odds of use of EVD in TICH patients with IVH, fourth ventricle compression, and acute hydrocephalus (20–24). Due to the special anatomical position of the cerebellum, it is easy to form acute obstructive hydrocephalus, and the compensation space is narrow, making the traumatic intracerebellar hemorrhage (TICH) become extremely dangerous. The patient's condition can change rapidly, even before the consciousness and pupil change, breathing and heartbeat can suddenly stop, giving no chance of rescue. Therefore, for TICH patients with admission CT showing IVH, fourth ventricle compression, and acute hydrocephalus, we prefer to place EVD as soon as possible before surgery. It is also important to note that, patients with intracerebellar hemorrhages just like all other severe TBI patients require intensive management meant to optimize blood glucose, base excess, mean arterial pressure, PaO_2_/FiO_2_ ratio, and serum hemoglobin, all known as relevant prognostic factors in this patients' group (25).

Previous studies showed that EVD use was associated with a trend toward decreased mortality but a greater modified Rankin Scale score for functional outcomes in spontaneous intracerebral hemorrhage (10). Similarly, our results demonstrated that placement of EVD significantly improved the neurological score at discharge and 6 months, regardless of the presence or absence of acute hydrocephalus. We believe that preoperative placement of EVD can improve the prognosis mainly in the following aspects: (1) Patients who need cerebellar hematoma evacuation generally have different degrees of intracranial hypertension. Preoperative EVD should be performed first to relieve intracranial hypertension and then hematoma evacuation. Otherwise, the cerebellum will become acutely inflated after cutting the dura mater, which has obvious negative effects on the surgical operation. (2) If EVD is performed after the hematoma is eliminated, the morphology of the lateral ventricle changes due to the outflow of cerebrospinal fluid, which increases the difficulty of successful puncture. (3) If the postoperative cerebellar edema is obvious and the hematoma remains, the continuous drainage by EVD can help patients through the peak period of edema.

Studies have shown that EVD can increase the risk of bleeding and infection in patients with traumatic brain injury. However, the current research has not found that preoperative EVD can increase the chance of bleeding and intracranial infection. Furthermore, the results showed a trend that EVD can decrease the occurrence of delayed hydrocephalus, which may be due to the continuous drainage of bloody cerebrospinal fluid by EVD, reducing subarachnoid adhesions.

## Conclusion

For TICH patients, perioperative EVD is safe and can significantly improve neurological prognosis. Especially for patients whose GCS dropped by more than 2 points before the operation, EVD can significantly improve the patient's GCS score, reduce the risk of herniation, and gain more time for surgical preparation. Even for TICH patients without acute hydrocephalus on admission CT scan, EVD placement still has positive clinical significance. However, future studies with larger sample sizes are warranted to confirm whether perioperative EVD can reduce in-hospital mortality and the risk of delayed hydrocephalus in TICH.

## Data availability statement

The raw data supporting the conclusions of this article will be made available by the authors, without undue reservation.

## Ethics statement

The studies involving human participants were reviewed and approved by Ethics Committee of Tangdu Hospital, Air Force Medical University (TDLL-202206-02). The patients/participants provided their written informed consent to participate in this study.

## Author contributions

X-LW, YQ, and BW proposed and designed this study. LG, S-nG, and Z-hL completed all the surgical operations. M-mS, GZ, HG, FG, Y-WS, and W-xC collected and organized the data. YW and WS performed the statistical analysis. BW and YZ were the major contributors to writing the manuscript. All authors read and approved the final manuscript.

## Funding

This work was supported by the National Natural Science Foundation of China (Grant No. 81801256) (BW), Shaanxi Provincial Association of Science and Technology Youth Talents Lifting Plan (Grant No. 20190302) (BW), Air Force Medical University Talent Support LingYun Project-Eagle Program (2019cyjhwb), Tangdu Hospital Youth Talent supporting Plan (BW), and Shaanxi Province Youth Science and Technology Rising Star Project (Grant No. 2017KJXX-13) (LG).

## Conflict of interest

The authors declare that the research was conducted in the absence of any commercial or financial relationships that could be construed as a potential conflict of interest.

## Publisher's note

All claims expressed in this article are solely those of the authors and do not necessarily represent those of their affiliated organizations, or those of the publisher, the editors and the reviewers. Any product that may be evaluated in this article, or claim that may be made by its manufacturer, is not guaranteed or endorsed by the publisher.

## Abbreviations

EVD: External ventricular drainage
HE: Hematoma evacuation
GCS: Glasgow coma scale
GOS: Glasgow Outcome Scale
TICH: Traumatic intracerebellar hematoma.

## References

[1] d'Avella D Servadei F Scerrati M Tomei G Brambilla G Angileri FF. Traumatic intracerebellar hemorrhage: clinicoradiological analysis of 81 patients. Neurosurgery. (2002) 50:16–25; discussion−7. 10.1227/00006123-200201000-00004 11844230

[2] Harsh V Prakash A Barry JM Kumar A. Traumatic intracerebellar haematoma: to operate or not to operate? Br J Neurosurg. (2015) 29:353–7. 10.3109/02688697.2014.987215 25488388

[3] D'Avella D Cacciola F Angileri FF Cardali S La Rosa G Germano A. Traumatic intracerebellar hemorrhagic contusions and hematomas. J Neurosurg Sci. (2001) 45:29–37. 11466505

[4] Takeuchi S Wada K Takasato Y Masaoka H Hayakawa T Yatsushige H. Traumatic hematoma of the posterior fossa. Acta Neurochir Suppl. (2013) 118:135–8. 10.1007/978-3-7091-1434-6_24 23564119

[5] Takeuchi S Takasato Y Masaoka H Hayakawa T. Traumatic intra-cerebellar haematoma: study of 17 cases. Br J Neurosurg. (2011) 25:62–7. 10.3109/02688697.2010.500410 20649395

[6] Hemphill JC 3rd Greenberg SM Anderson CS Becker K Bendok BR Cushman M. Guidelines for the management of spontaneous intracerebral hemorrhage: a guideline for healthcare professionals from the American Heart Association/American Stroke Association. Stroke. (2015) 46:2032–60. 10.1161/STR.0000000000000069 26022637

[7] Mitchell P Gregson BA Vindlacheruvu RR Mendelow AD. Surgical options in ich including decompressive craniectomy. J Neurol Sci. (2007) 261:89–98. 10.1016/j.jns.2007.04.040 17543995

[8] Carney N Totten AM O'Reilly C Ullman JS Hawryluk GW Bell MJ. Guidelines for the management of severe traumatic brain injury, fourth edition. Neurosurgery. (2017) 80:6–15. 10.1227/NEU.0000000000001432 27654000

[9] Sumer MM Acikgoz B Akpinar G. External ventricular drainage for acute obstructive hydrocephalus developing following spontaneous intracerebral haemorrhages. Neurol Sci. (2002) 23:29–33. 10.1007/s100720200020 12111618

[10] Lovasik BP McCracken DJ McCracken CE McDougal ME Frerich JM Samuels OB. The effect of external ventricular drain use in intracerebral hemorrhage. World Neurosurg. (2016) 94:309–18. 10.1016/j.wneu.2016.07.022 27436212

[11] Gardner PA Engh J Atteberry D Moossy JJ. Hemorrhage rates after external ventricular drain placement. J Neurosurg. (2009) 110:1021–5. 10.3171/2008.9.JNS17661 19199471

[12] Dorresteijn K Jellema K van de Beek D Brouwer MC. Factors and measures predicting external Csf drain-associated ventriculitis: a review and meta-analysis. Neurology. (2019) 93:964–72. 10.1212/WNL.0000000000008552 31659095

[13] Champey J Mourey C Francony G Pavese P Gay E Gergele L. Strategies to reduce external ventricular drain-related infections: a multicenter retrospective study. J Neurosurg. (2018) 1:1–6. 10.3171/2018.1.JNS172486 29932377

[14] Kothari RU Brott T Broderick JP Barsan WG Sauerbeck LR Zuccarello M. The abcs of measuring intracerebral hemorrhage volumes. Stroke. (1996) 27:1304–5. 10.1161/01.STR.27.8.1304 8711791

[15] Chung DY Olson DM John S Mohamed W Kumar MA Thompson BB. Evidence-based management of external ventricular drains. Curr Neurol Neurosci Rep. (2019) 19:94. 10.1007/s11910-019-1009-9 31773310 PMC7383112

[16] Chung DY Mayer SA Rordorf GA. External ventricular drains after subarachnoid hemorrhage: is less more? Neurocrit Care. (2018) 28:157–61. 10.1007/s12028-017-0443-2 28929378 PMC5858985

[17] Thabet AM Kottapally M Hemphill JC 3rd. Management of intracerebral hemorrhage. Handb Clin Neurol. (2017) 140:177–94. 10.1016/B978-0-444-63600-3.00011-8 28187799

[18] Dey M Jaffe J Stadnik A Awad IA. External ventricular drainage for intraventricular hemorrhage. Curr Neurol Neurosci Rep. (2012) 12:24–33. 10.1007/s11910-011-0231-x 22002766 PMC6777952

[19] Herrick DB Ullman N Nekoovaght-Tak S Hanley DF Awad I LeDroux S. Determinants of external ventricular drain placement and associated outcomes in patients with spontaneous intraventricular hemorrhage. Neurocrit Care. (2014) 21:426–34. 10.1007/s12028-014-9959-x 24522761

[20] Jaffe J Melnychuk E Muschelli J Ziai W Morgan T Hanley DF. Ventricular catheter location and the clearance of intraventricular hemorrhage. Neurosurgery. (2012) 70:1258–63; discussion 63–4. 10.1227/NEU.0b013e31823f6571 22067423 PMC3719169

[21] Kakarla UK Kim LJ Chang SW Theodore N Spetzler RF. Safety and accuracy of bedside external ventricular drain placement. Neurosurgery. (2008) 63(1 Suppl 1):ONS162-6; discussion ONS6-7. 10.1227/01.NEU.0000312390.83127.7F 18728595

[22] Hanak BW Walcott BP Codd PJ Jones PS Nahed BV Butler WE. Fourth ventricular neurocystercercosis presenting with acute hydrocephalus. J Clin Neurosci. (2011) 18:867–9. 10.1016/j.jocn.2010.12.002 21507657

[23] Roitberg BZ Khan N Alp MS Hersonskey T Charbel FT Ausman JI. Bedside external ventricular drain placement for the treatment of acute hydrocephalus. Br J Neurosurg. (2001) 15:324–7. 10.1080/02688690120072478 11599448

[24] Muralidharan R. External ventricular drains: management and complications. Surg Neurol Int. (2015) 6:S271–4. 10.4103/2152-7806.157620 26069848 PMC4450504

[25] Prisco L Iscra F Ganau M Berlot G. Early predictive factors on mortality in head injured patients: a retrospective analysis of 112 traumatic brain injured patients. J Neurosurg Sci. (2012) 56:131–6. 22617175

